# Ecosystem services valuation for supporting sustainable life below water

**DOI:** 10.1186/s42055-023-00068-1

**Published:** 2023-12-20

**Authors:** Phoebe Koundouri, George Halkos, Conrad Felix Michel Landis, Angelos Alamanos

**Affiliations:** 1 School of Economics, Athens University of Economics and Business, Athens, Greece; 2 Department of Technology, Management and Economics, Denmark Technical University (DTU), Kongens Lyngby, Denmark; 3 Sustainable Development Unit, Athena RC, Athens, Greece; 4 Academia Europea, Athens, Greece; 5 ReSEES Research Laboratory, Athens University of Economics and Business, Athens, Greece; 6 Department of Economics, University of Thessaly, Volos, Greece; 7 Visiting Faculty, Indian Institute of Management Rohtak, Rohtak, India

**Keywords:** Sustainability, SDG14, Environmental valuation, Ecosystem services, Sustainability reporting frameworks

## Abstract

The significance of the SDGs lies in their holistic, global and interdisciplinary nature. But this nature at the same time poses significant challenges, as it is difficult to bridge the breadth of different aspects included in the SDGs, such as the environmental and the socio-economic, both in theory, practical application and policymaking. SDG14 on “life below water” is quite a holistic concept as it refers to a natural/environmental system (seas), supporting several marine economic activities and ecosystem values, and associated with strong social and cultural characteristics of the local populations, affecting the ways they manage marine areas. The main challenges for the achievement of a sustainable life below water are analyzed, and ways forward are discussed. Holistic and well-coordinated approaches considering the complex nature of SDG14 are necessary. Moreover, we argue on the role of economic instruments that can bridge environmental and socio-economic aspects, towards more sustainable life below water. In particular, the potential of environmental valuation as a means to better inform SDG policies, is discussed, using the example of SDG14. The currently established frameworks for Country’s Sustainability Reporting, lack metrics focusing on the economic impact of the environment and the ecosystem services’ degradation or restoration rates, including ocean and marine ecosystems. Acknowledging and quantifying the costs and benefits of ocean and marine ecosystems can lead to more effective interventions (such as ocean pollution prevention, climate change mitigation, fishing exploitation, biodiversity and coral reef preservation) and a better understanding of human-environmental dynamics. This, in turn, strengthens coordinated management and cooperation.

## Introduction

The need for sustainable ways of development is widely acknowledged in science and literature, as the world increasingly calls for resilience and integrity in various aspects, such as the economy, society, and the natural environment. These three aspects are connected with the concept of sustainability (‘pillars’ of sustainability [1]), and can be extended and deepened (e.g. to several diverse environmental systems, or economic processes), and simultaneously, affect the ways that such systems interact (i.e., intersections of economic, environmental, social, cultural spaces) at national and international scales [2]. So, sustainable development can be seen as a phenomenon of our modern world that brings together various processes and disciplines, in a broader space to provide solid solutions to global problems, such as climate change, resource scarcity and depletion, inequalities, and well-being.

Sustainable development needs a balancing course of well-organized action to equilibrate its pillars and the disciplines involved, given its inclusive nature [3]. The United Nations’ (UN) 2030 Agenda with the 17 Sustainable Development Goals (SDGs) and the 169 associated targets serve currently as such a course of action, a pathway that can lead to a sustainable world. The significance of the SDGs lies in their global commitment by all UN member countries and their holistic, interdisciplinary approach to addressing pressing global challenges. However, the very attributes that make the SDGs powerful (global and interdisciplinary nature), also pose significant challenges to their achievement, as we need commitment from all the relevant disciplines. Policies need to consider this need and create bridges among the scientific fields involved in each SDG.

In this paper we use SDG14 “Life Below Water” as an example that clearly reflects this integrated nature: It combines the environmental component (oceans, seas, marine resources) and the interconnected socio-economic system with the associated activities and well-being. We argue that the current approaches often miss this integrated character by overlooking the socio-economic ad cultural aspects of SDG14, and we discuss how scientific instruments considering these aspects can contribute to a more sustainable life below water. The paper is organized as follows: The necessary background information is provided regarding Environmental Economics and valuation; next SDG14 is described and its main challenges are analysed; A way forward is presented with our opinion for the need of more holistic approaches and the integration of economic instruments for overcoming the challenges for the achievement of SDG14.

## The role of Environmental Economics and valuation

Ecosystems provide essential services that enhance the quality of life for all entities. These services, including regulation, provisioning, cultural, and support functions, constitute the benefits derived from ecosystems (Ecosystem Services – ES). Since most ES lack market prices, assigning them monetary values is challenging [4]. Environmental valuation studies assign a monetary measure of the benefit or cost to the welfare status of individuals and social groups regarding improvement interventions or the impacts of environmental degradation [5, 6]. These insights help policymakers prioritize and manage ES effectively or allocate environmental and economic resources more efficiently to maximize economic, social, and environmental gains while preserving ecological integrity [7, 8].

Environmental valuation studies have their roots in the concept of Total Economic Values, which are divided into two major sub-categories: use values (direct, indirect, and option values) and nonuse values (existence and bequest values). In this context, environmental valuation offers various techniques for assigning monetary values to environmental impacts/changes, such as stated preference methods and revealed preference methods [9]: Stated preference methods (e.g. choice experiments and contingent valuation methods) involve hypothetical scenarios and questions to gauge individuals’ preferences, while revealed preference methods observe real behavior. Choice experiments present alternatives and attributes related to an environmental good or service [10]. Contingent valuation studies focus on estimating people’s Willingness-To-Pay or Willingness-To-Accept for environmental quality changes [11, 12]. Revealed preference methods analyze actual behavior to estimate the value of ecosystem benefits, helping assess the costs and benefits associated with pollution, noise, aesthetics, and proximity to recreational sites.

Environmental valuation can play a pivotal role in advancing the objectives of SDG14, by valuing ES associated with marine environments and their natural capital (resources, materials, fishing), showing the economic impacts and relations of human activities on marine ecosystems (tourism, conservation, recreation, aesthetic values). The knowledge of these values aligns with SDG14’s targets to become more efficient in preventing and reducing marine pollution, sustainably manage and protect marine and coastal ecosystems, and regulate overfishing. Through techniques like contingent valuation and choice experiments, we can capture societal preferences regarding marine conservation and quantify the economic benefits of maintaining healthy oceans. This information is crucial for policymakers in prioritizing interventions, allocating resources, and designing effective strategies to achieve SDG14, fostering a balanced approach that harmonizes economic development with the preservation of life below water.

As mentioned, monetizing ES has weaknesses, as not all values can be quantified in monetary terms, or this is at least challenging. This is one limitation of this approach. It should be made clear that assigning financial values may oversimplify complex ecological relationships, leading to undervaluation or neglect of certain services [13], limiting the effectiveness of the relevant management efforts. Market-based approaches can also neglect vulnerable communities, potentially exacerbating social inequalities [14, 15].

## SDG14 and challenges to its achievement

SDG14 focuses on protecting marine ecosystems, reducing marine pollution, addressing overfishing, and promoting the sustainable management of coastal and marine areas to ensure the well-being of both marine life and human communities that depend on them, socially and economically. Its key performance indicators (KPIs) refer to the protected areas for biodiversity conservation; the “ocean health” (clean waters, not contaminated by chemicals, nutrients, human pathogens, and trash, avoid ocean acidification); marine-biodiversity-threats; fishing exploitation; and the crucial role of oceans in climate regulation. The UN’s Department of Economic and Social Affairs and the relevant Statistics Division consider 10 indicators representing relevant metrics to these KPIs to measure progress for SDG14. The literature is rich of examples that have used environmental valuation methods for SDG14 – related applications, including the coastal cultural ES [16], biodiversity protection [17], and many more [18].

From the economic point of view, most KPIs could be informed and significantly enhanced by translating them into monetary terms, as oceans and marine ecosystems are full of ES. They provide food through fisheries, regulate climate by absorbing carbon dioxide, and generate oxygen. They also support cultural and recreational activities, such as tourism and spiritual practices, while serving as transportation routes and habitats for diverse marine life [19]. Additionally, oceans contribute to scientific knowledge, offering valuable insights into climate dynamics and biodiversity [17]. Their role in coastal protection, by buffering against storms and erosion, further underscores their significance [18]. These indicative ES, clearly have use and nonuse values, as defined in the previous section. However, this contribution is not well-recognized, and is often neglected when management interventions are designed. The achievement of SDG14 globally has been characterized as “a round and inclusive failure”, for a plethora of reasons [20]. Table 1 summarizes the main threats for a sustainable “life below water”.

**Table 1:** The main challenges for the achievement of SDG14

**Challenge**	**Description**
Ocean Pollution	Pollution from various sources, including plastic waste, industrial discharges, and agricultural runoff, poses a significant threat to marine ecosystems, harming marine life, disrupting ecosystems, and affecting human health [21].
Climate Change Impacts	Climate change is leading to rising sea levels, ocean acidification, and altered oceanic currents. These changes affect marine ecosystems and the communities that rely on them [22].
Global Cooperation	There are insufficient global governance frameworks and cooperation to address transboundary issues. Conflicts over maritime boundaries and resources, or unequal rights, hinder progress [20].
Lack of coordinated management	Effective and coordinated management of marine resources across different sectors (e.g., fisheries, shipping, tourism) is often lacking, and there is often a competition of users to generate benefits from marine ecosystems [23].
Overfishing and Depleting Fish Stocks	Many regions are experiencing unsustainable fishing practices, which threaten fish stocks’ viability, marine ecosystems, and the livelihoods of coastal communities [24].
Illegal, Unreported, and Unregulated (IUU) Fishing	IUU fishing remains a major problem, as it undermines efforts to conserve marine resources and enforce regulations. It leads to unfair competition, environmental degradation, and economic losses [24].
Coral Reef Decline	Coral reefs, which are critical for marine biodiversity, are under threat from rising sea temperatures, ocean acidification, and physical damage from human activities. Protecting and restoring coral reefs is a pressing challenge [17].
Biodiversity Collapse	The loss of marine biodiversity due to habitat destruction, pollution, and climate change is a grave concern [25].
Inadequate Data and Monitoring	Gathering comprehensive data on the state of the oceans and the impact of policies and actions is challenging, and there is very limited progress on this along several environmental SDGs, making accurate progress-tracking challenging [26].
Resource Constraints	Many coastal and developing countries lack the financial and technical resources to implement sustainable ocean management practices effectively [27].
Community-management understanding	There is limited understanding of community-based marine management approaches, as they are under-studied. Successful examples are difficult to generalize, as they are subject to local-specific and cultural factors [15, 28].

The multidisciplinary character of scientific efforts to simulate and improve the natural-human marine systems is also a challenge. The challenges mentioned in Table 1 are diverse, and are not always comparable (e.g.in terms of spatial extent), or subject to the same metrics (or even units of measurement), so they cannot be tackled with the same interventions. Moreover, each one of these challenges can be highly case-specific, as they are strongly connected with each area’s natural, social, economic and cultural characteristics. Haas [29] indicate that despite its importance, SDG14 is one of the least studied and most under-implemented SDGs, given its highly case- and region-specific character across different countries and income groups.

## Conclusion - the way forward

We believe that challenges in achieving SDG14 are interconnected with broader issues that frequently impede progress towards sustainable development. These issues can include a lack of integrated management across different actors and uses, which is reflected by the limited studies on SDG14, and a poor understanding of its context.

SDG14 exhibits significant interconnections among its sub-goals and indicators with other SDGs [30, 31]. Researchers in the field are investigating trade-offs between SDG14 and other SDGs, as documented in the relevant literature [32, 33]. One issue that needs further consideration is the trade-offs between SDG14 and other SDGs from the lens of distributive and procedural justice [34]. At a managerial level, Ntona and Morgera [35] note the contribution of effective marine spatial planning to interrelate SDG14 with other SDGs and collectively move toward an “environment for well-being” approach under the Convention on Biological Diversity. Improving the SDGs needs holistic and effective management, based on scientifically-supported interventions.

From the Economics point of view, we believe that environmental valuation should be used to inform SDG-related policies. Policy measures are evaluated based on their expected costs and benefits, so the insights of environmental valuation studies can be used as inputs at the stage of the cost-benefit analysis of the interventions under consideration. Matching environmental valuation studies with SDGs creates many research opportunities. Indicatively, for any environmental-related SDG (e.g. inclusive growth, climate action, clean water, life below water, life on land, etc.) environmental valuation methods can be employed, assessing different development efforts, implementation steps (e.g., use of resources), and monitor progress achieved (e.g., environmental indicators and benchmarking targets) when managing or exploiting natural resources (e.g., preserve, conserve, restore, enjoy). For the example of SDG14, the ES of marine and coastal ecosystems should be embedded in the decision-making process and the assessment of any centralized measures. These ES cover most aspects of SDG14, as they include provisioning services (fisheries and raw materials); supporting services (life-cycle maintenance for both fauna and local, element and nutrient cycling); regulating services (climate, carbon sequestration and storage, erosion prevention, waste-water treatment, moderation of extreme events); and cultural services (tourism, recreational, aesthetic, and spiritual benefits).

Acknowledging and quantifying the costs and the benefits of each one of the challenges outlined in Table 1 will lead to more effective interventions (e.g. on ocean pollution prevention, climate change mitigation, fishing exploitation, biodiversity and coral reefs preservation). Better understanding of the human-environmental dynamics as reflected from valuation studies can serve to strengthen coordinated management and cooperation, along with the data and monitoring efforts, which are necessary to perform such studies.

The integration of environmental valuation into the cost-benefit analyses of interventions also reflect the relationships between social welfare and the environment, which are not static. They evolve as new challenges occur (e.g., climate change and climate crisis), new consumptive and spending patterns appear, and change as core determinants of demand and supply differentiate and alter according to natural resource depletion patterns. According to Koundouri et al. [36], societies attributing greater value to ES mark greater progress toward the implementation of SDGs and SDG 14 in particular, as high Willingness-To-Pay indicates behavioral changes that leads to higher implementation of SDGs. These issues highlight the significance of valuation studies not only as a subject of economic theory but also as a means of recognizing interdependencies among social and environmental factors within the economic system. After all, this is the critical idea of ecosystem valuation: to unravel the complexities of socio-ecological relationships, make clear how human decisions affect ES values, and direct these value changes in monetary units to facilitate their inclusion in public decision-making processes. Based on the above considerations, the ecosystem valuation estimates could be integrated in the decision-making tools such as the SDG Indicators [37].

As mentioned, this approach is accompanied by inherent weaknesses, which must be taken into account to avoid negative consequences and mismanagement (e.g. oversimplification, missing certain ecosystem values, potentially subjective nature, inequality concerns). We believe that monetizing ecosystem services can work under certain conditions, such as clear valuation methods, incorporating both economic and non-economic factors (addressing non-monetary considerations), community engagement (ensuring that diverse perspectives are considered, and mitigating social inequality concerns). Tailoring approaches to specific contexts and ecosystems are crucial. Effective governance, including transparent decision-making and adaptive management, helps address complexities. A balanced approach, acknowledging both monetary and non-monetary values, can foster comprehensive management of human – marine ecosystems.

These efforts should concern interregional, national, and international research attempts aligned to SDGs, and especially in SDG14. In other words, all econometric models should consider all pillars of sustainability to reap benefits in terms of theoretical and practical implications. Researchers should employ various determinants and proxies of environmental quality, social and human well-being, and high-leverage market segments to accomplish this task. Additionally, comparative studies will offer a lot to understand relevant interdependencies and interrelations under different econometric schemes and approaches. Last but not least, since all these concepts and notions described above synthesize a dynamic and complex system, scientists ought to communicate among them and to policymakers the relevant research findings regularly. This is an advantageous and dependable way to move forward faster and safer towards a better world.

## Data Availability

UN, United Nations - Department of Economic and Social Affairs (2023). Sustainable Development, https://sdgs.un.org/goals.

## Abbreviations

SDGs: Sustainable Development Goals
ES: Ecosystem Services
KPIs: Key Performance Indicators
